# 4‐1BB costimulation promotes bystander activation of human CD8 T cells

**DOI:** 10.1002/eji.202048762

**Published:** 2020-12-23

**Authors:** Manuel Reithofer, Sandra Rosskopf, Judith Leitner, Claire Battin, Barbara Bohle, Peter Steinberger, Beatrice Jahn‐Schmid

**Affiliations:** ^1^ Department of Pathophysiology and Allergy Research Center for Pathophysiology Infectiology and Immunology Medical University of Vienna Vienna Austria; ^2^ Division of Immune Receptors and T Cell Activation Institute of Immunology Center for Pathophysiology Infectiology and Immunology Medical University of Vienna Vienna Austria

**Keywords:** Bystander activation, CD27, CD8, Costimulation agonist, T‐cell costimulation

## Abstract

Costimulatory signals potently promote T‐cell proliferation and effector function. Agonistic antibodies targeting costimulatory receptors of the TNFR family, such as 4‐1BB and CD27, have entered clinical trials in cancer patients. Currently there is limited information how costimulatory signals regulate antigen‐specific but also bystander activation of human CD8 T cells.

Engineered antigen presenting cells (eAPC) efficiently presenting several common viral epitopes on HLA‐A2 in combination with MHC class I tetramer staining were used to investigate the impact of costimulatory signals on human CD8 T‐cell responses. CD28 costimulation potently augmented the percentage and number of antigen‐reactive CD8 T cells, whereas eAPC expressing 4‐1BB‐ligand induced bystander proliferation of CD8 T cells and massive expansion of NK cells. Moreover, the 4‐1BB agonist urelumab similarly induced bystander proliferation of CD8 T cells and NK cells in a dose‐dependent manner. However, the promotion of bystander CD8 T‐cell responses is not a general attribute of costimulatory TNF receptor superfamily (TNFRSF) members, since CD27 signals enhanced antigen‐specific CD8 T cells responses without promoting significant bystander activation.

Thus, the differential effects of costimulatory signals on the activation of human bystander CD8 T cells should be taken into account when costimulatory pathways are harnessed for cancer immunotherapy.

## Graphical abstract

Using a novel system of engineered antigen presenting cells (eAPC), we demonstrate that 4‐1BB signals generated by 4‐1BBL or agonistic 4‐1BB antibodies promote bystander activation of human CD8 T cells

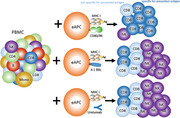

## Introduction

CD8 T cells are prime targets for immunotherapeutic interventions against tumors and viruses. Such strategies include the ex vivo expansion of antigen‐specific cytotoxic T lymphocytes (CTLs) or the generation of autologous CD8 T cells genetically retargeted toward malignant or virus‐infected cells, as well as the administration of immunostimulatory antibodies to improve T‐cell responses to tumors in vivo. The success of such approaches greatly depends on the availability of sufficient and appropriate activating signals to CTLs targeting antigens of interest. Costimulatory signals have received much attention in this context since their presence is a prerequisite for the efficient responses of T cells activated via their TCR complex. They can counteract T‐cell exhaustion and regulate activation‐induced cell death (AICD), which is associated with the clonal expansion of T cells [1, 2, 3, 4, 5, 6]. However, costimulatory signals have to be tightly regulated, since they have the potential to break self‐tolerance and mediate aberrant and harmful immune responses. Therefore, interventions aimed at introducing such signals bear the danger of serious side effects. Costimulatory receptors belong to distinct families of molecules and exhibit qualitative differences in the signals that they induce upon engagement. CD28 is generally regarded as the primary costimulatory receptor on T cells but several additional molecules, including 4‐1BB, OX40, CD27, ICOS, and CD2, are able to transduce strong activating signals into T cells engaged in antigen recognition [7, 8, 9]. A great deal of research has focused on 4‐1BB, which is often regarded as the most important cosignal for antigen‐experienced CD8 T cells, and which has been previously found to efficiently mediate the expansion and acquisition of effector function in this subset [10, 11, 12, 13, 14]. 4‐1BB signaling promotes the expression of IL‐2 and IFN‐γ and induces survival molecules such as Bcl‐xL and BFL‐1 [10, 15, 16]. In addition, 4‐1BB engagement enhances mitochondrial mass and respiratory capacity, thereby promoting the metabolic reprogramming of T cells during activation [17]. Hypoxia, which is invariably observed in the tumor microenvironment, induces 4‐1BB upregulation such that tumor‐infiltrating lymphocytes (TILs) may be a preferable target for the agonistic antibodies targeting this receptor [18].

Much of our current view on the regulation of T‐cell responses is based on murine studies. The availability of TCR‐transgenic models as well as mice deficient in distinct costimulatory pathways, has facilitated studies on the impact of accessory signals on antigen‐specific T‐cell responses. By contrast, studies on the role of accessory signals in human T cells have mainly focused on bulk T cells or T‐cell subsets stimulated in an antigen‐independent manner, for example, by CD3 antibodies or antibody fragments [10, 12, 19, 20]. Although both approaches have yielded invaluable insights into the relationship between accessory signals and T‐cell responses, they do not fully reflect how the response of human T cells activated by cognate antigen is regulated by cosignals. Studies using antigen‐specific human T cells, however, are impeded by the scarcity of T cells specific to a certain antigen in a given donor. Moreover, the activation of T cells with antigens presented by natural APC like DC harboring numerous different accessory molecules results in the engagement of several costimulatory receptors, which makes it difficult to discern the role of individual pathways in T‐cell activation and reprograming processes.

In this study, we established an experimental system that overcomes both obstacles. We devised engineered antigen presenting cells (eAPC), which endogenously express and efficiently present multiple major viral epitopes in the context of HLA‐A*02:01, a globally common HLA type. These K562‐based cells induced antigen‐specific CD8 T‐cell responses in the large majority of PBMC‐samples derived from HLA‐A2 positive donors. Using these eAPC, we studied the impact of major costimulatory ligands on the antigen‐specific human CD8 T‐cell response in vitro. In addition, we exploited our eAPC platform to assess the effects of costimulation‐agonists which are currently evaluated in clinical trials regarding their safety and efficacy in cancer patients.

## Results

### Generation and validation of eAPC

**Figure 1:**
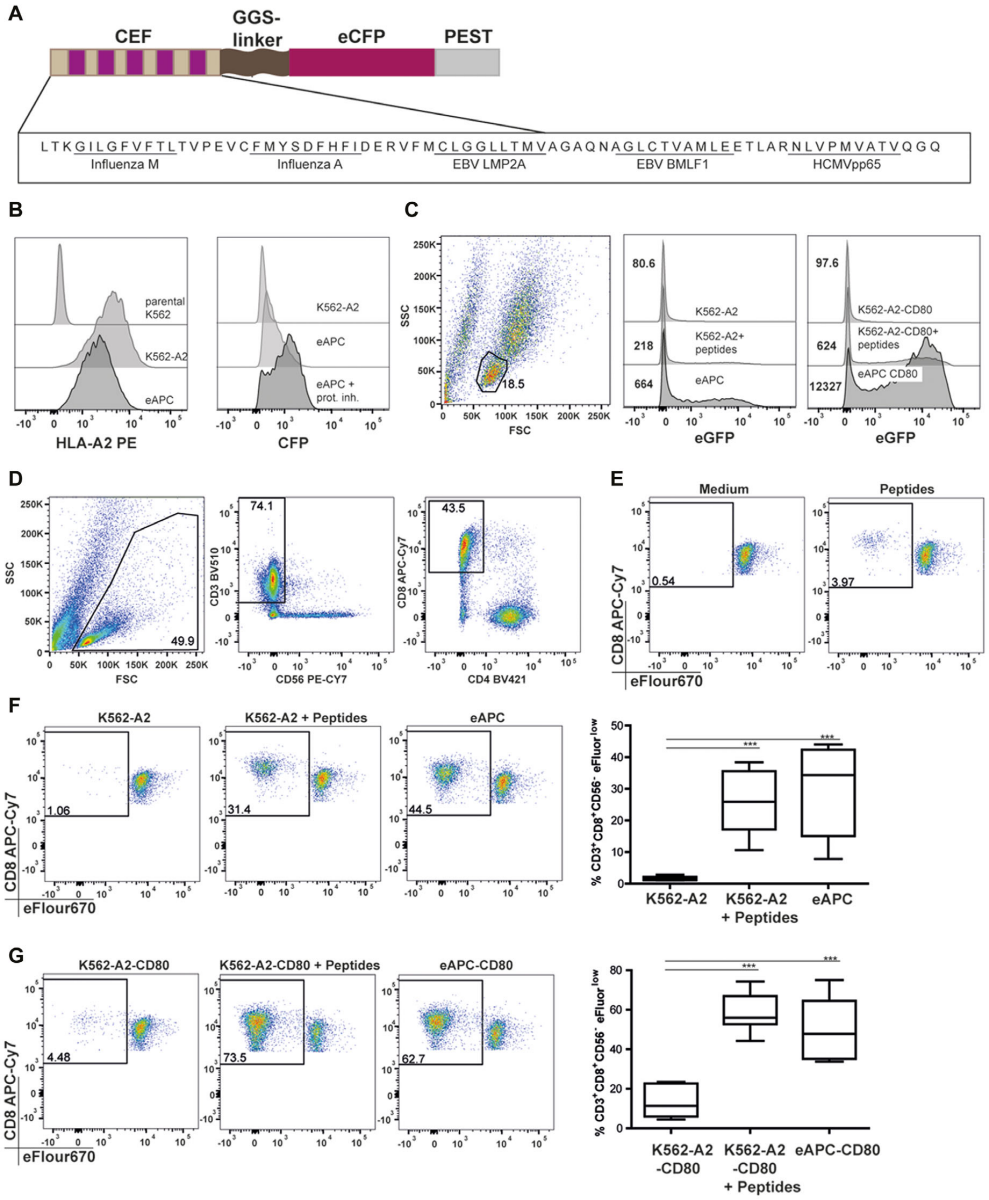
Generation and evaluation of eAPC. (A) Schematic representation of an antigen‐expression construct encoding five major HLA‐A2 restricted, viral T‐cell epitopes fused to eCFP and a sequence rich in proline, glutamic acid, serine, and threonine (PEST). (B) Left: K562 cells expressing HLA‐A2 (K562‐A2) and K562 cells coexpressing HLA‐A2 and the antigen‐expressing construct (eAPC) were probed with a HLA‐A2 specific antibody. The parental K562 line was used as a negative control. Right: eCFP expression on untreated eAPC and eAPC treated with a proteasome inhibitor. The eCFP‐negative K562‐A2 cells were used as a negative control. Numbers indicate gMFI. One representative out of three experiments is shown. Gating strategy is shown in Supporting information Fig. S1A. (C) CMVpp65‐TCRtg Je6 NF‐κB::GFP‐reporter cells were cocultured with the indicated cells lines for 16 h and analyzed for reporter gene expression. One of three independent experiments is shown. Numbers indicate gMFI. (D) Gating strategy used in this study to evaluate the proliferation of CD8 T cells in eFluor670‐labelled PBMC after stimulation for 1 week. Proliferation of CD8 T cells in response to (E) medium alone or viral peptides, (F) K562‐A2, K562‐A2 plus viral peptides, eAPC, and (G) proliferation of CD8 T cells in response to K562‐A2 or eAPC expressing CD80. (D‐G) One representative experiment and box‐and‐whisker blots (range minimum to maximum) representing cumulative data with samples from eight different donors from three independent experiments using two to three different PBMC donors are shown. One‐way ANOVA followed by Bonferroni post‐test; ***p ≤ 0.001. All data were measured by flow cytometry.

We generated a synthetic gene encoding a polypeptide containing five viral epitopes commonly recognized by HLA‐A2 positive donors fused to the fluorescent protein eCFP and a PEST sequence for efficient proteasome targeting and antigen processing (Fig. 1A). This construct was expressed in the human erythroleukemia cell line K562 engineered to express homogeneous levels of HLA‐A2. The resulting cell lines (eAPC) were analyzed by flow cytometry (Supporting information Fig. S1). The addition of a proteasome inhibitor strongly enhanced eCFP expression indicating efficient proteasomal targeting of the antigenic polypeptide (Fig. 1B). To confirm presentation of antigenic peptides on the eAPC they were cocultured with transcriptional Jurkat E6.1 NF‐κB::eGFP reporter cells expressing a TCR specific for the peptide NLVPMVATV, derived from the CMV protein pp65 [21], one of the antigens expressed in the eAPC. The reporter activation induced upon coculture with eAPC was greater compared to K562‐HLA‐A2 cells exogenously loaded with peptides. The presence of the costimulatory CD28 ligand CD80 markedly increased this reporter activation (Fig. 1C).

Thereupon, eAPC were tested in proliferation experiments for their capacity to induce T‐cell proliferation in eFluor670‐labelled PBMCs derived from healthy HLA‐A2 positive donors. The sole addition of exogenous viral peptides to PBMCs only weakly stimulated T‐cell proliferation, as indicated by the low percentage of eFluor670^low^ CD8 T cells observed after 6 days of stimulation (Fig. 1D and E). The coculture of PBMCs with K562‐A2 plus exogenous peptides or eAPC resulted in a significantly increased CD8 T‐cell response (Fig. 1F). Moreover, the presence of the costimulatory ligand CD80 on peptide‐loaded K562‐HLA‐A2 or eAPC significantly enhanced the percentages of proliferated CD8 T cells in the stimulation cultures (Fig. 1G). Time course experiments revealed that eFluor670^low^ CD8 T cells were already detectable in day 3 cocultures, which gradually increased until day 7 (Supporting information Fig. S2).

### eAPC induce potent antigen‐specific proliferation and effector function

**Figure 2:**
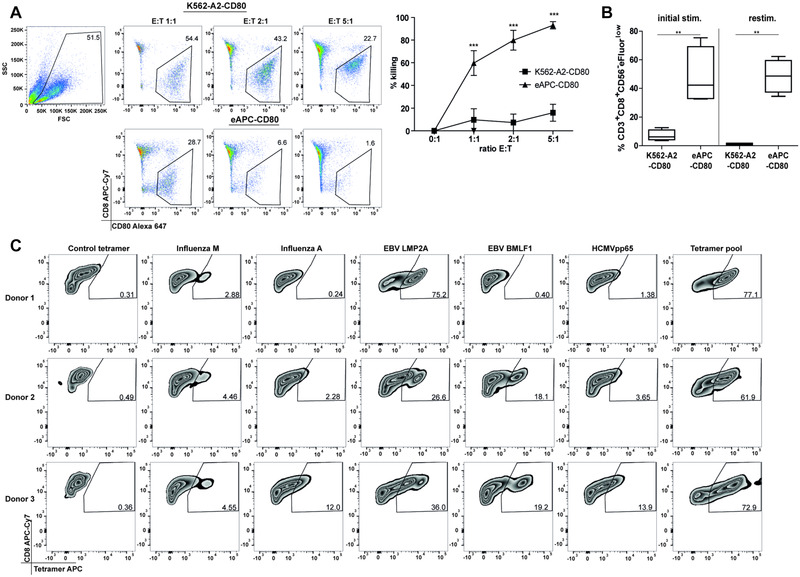
eAPC‐CD80 induce potent antigen‐specific proliferation and effector function. (A) Cytotoxic activity of sorted, CSFE^low^ CD8 T cells obtained after 7 days of stimulation with eAPC. Gating strategy to identify life cells and dot plots of one representative donor out of three analyzed in different experiments. (mean + SEM). One‐way ANOVA and Bonferroni post‐test (***p ≤ 0.001) was used for statistical analysis. (**B**) Proliferation of CD8 T cells after initial stimulation of PBMC from five different donors analyzed in independent experiments with either K562‐A2 or eAPC (left) and after restimulation of eAPC‐stimulated cells with K562‐A2 or eAPC are shown as box‐and‐whisker blots (range minimum to maximum). Statistical analysis was performed using one‐way ANOVA followed by Bonferroni post‐test (**p ≤ 0.01; ***p ≤ 0.001) was performed. (**C**) PBMCs from three different HLA‐A2 positive donors were stimulated with eAPC and pHLA‐A2 specificity of CSFE^low^ CD8 T cells were analyzed with the indicated tetramers in three independent experiments (one donor per experiment). All data were measured by flow cytometry.

To assess the capability of eAPC‐stimulated CD8 T cells to exert effector function, PBMCs of HLA‐A2 positive donors were cocultured for 7 days with eAPC‐CD80. Subsequently proliferated CD8 T cells were isolated by flow‐sorting and cocultured at different effector to target ratios with eAPC‐CD80 or K562‐HLA‐A2‐CD80 for 10 h. Flow cytometric analysis revealed that stimulation with eAPC induced potent effector functions. The peptide‐presenting target cells were efficiently killed, whereas cytotoxicity toward control cells presenting no viral antigens was low (Fig. 2A). Moreover, the CD8 T cells that had proliferated in the primary stimulation cultures were found to vigorously proliferate in response to restimulation in a strictly antigen‐dependent manner (Fig. 2B).

Staining of eAPC‐stimulated PBMC with appropriate pHLA‐A2 tetramers after 7 days demonstrated that different donors responded to different antigenic peptides. Importantly, the large majority (median 79.5, range 61.9‐96.3, n = 13) of CFSE^low^ proliferated CD8 T cells were found to be stained positive with a pool of five pHLA‐A2 tetramers representing the five antigenic peptides presented by the eAPC (Fig. 2C). Taken together, these results demonstrate that eAPC induced potent antigen‐specific proliferation and effector function in our in vitro culture system.

### eAPC expressing 4‐1BBL promote bystander CD8 T‐cell responses and NK cell proliferation

**Figure 3:**
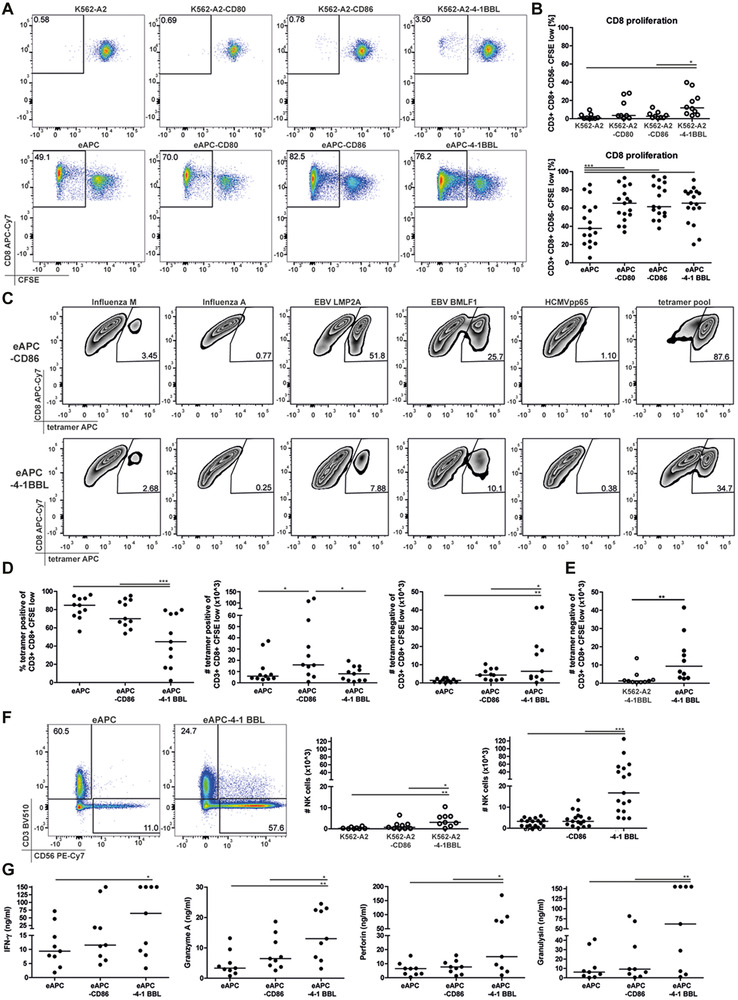
eAPC expressing 4‐1BBL promote bystander CD8 T‐cell responses and NK‐cell proliferation. (A) Proliferation of CD8 T cells of a representative donor in response to stimulation with the indicated K562‐A2 cell lines (upper panels) or with control‐eAPC and eAPC expressing the costimulatory molecules CD80, CD86, or 4‐1BB (lower panels). (B) CD8 proliferation of CD8 T cells in response to the indicated K562‐A2 cells (upper graph) or to the indicated eAPC (lower graph). The dots represent the proliferation of CD8 T cells from individual donors in response to stimulation with the indicated eAPC. Data from 10 and 17 donors (from six independent experiments with one to three donors) are shown in the upper and lower graphs, respectively. (C) Experiment showing antigen specificity of proliferated, that is, eFlour670^low^ CD8 T cells, after stimulation with eAPC‐CD80 (upper panel) or eAPC‐4‐1BBL (lower panel) determined by staining with indicated pHLA‐A2 tetramers and with a pool of all five pHLA‐A2 tetramers (representative for samples of 10 different donors analyzed in six independent experiments with one to two donors). (D) The samples of 10 different donors (from six independent experiments with one to two donors) were analyzed in independent experiments with a pHLA‐A2 tetramer pool. The percentages of tetramer positive (left graph), the absolute numbers of tetramer positive (middle), and tetramer negative (right) was determined in the proliferated CD8 T cells following stimulation with the indicated eAPC for 7 days. (E) Counts of tetramer‐negative proliferated CD8 cells upon stimulation with K562‐A2‐41BBL and eAPC‐41BBL. Data from 11 donors (from five independent experiments with one to three donors) are shown. (F) Left: Representative dot plots showing the percentages of CD3 negative/CD56 positive cells after stimulation with eAPC and eAPC 4‐1BBL. Right: Numbers of CD3 negative/CD56 positive cells in stimulation cultures with the indicated K562‐A2 cell lines not expressing viral antigens and the corresponding eAPC are shown. Data from nine donors (five independent experiments with one to two donors) and 17 donors (eight independent experiments with one to three donors) are shown for the K562‐A2 cell lines and eAPC cell lines, respectively. (G) IFN‐γ, granzyme A, perforin, and granulysin concentrations were determined in the supernatants of PBMC from nine different donors (five independent experiments with one to two donors) cocultured with eAPC‐CD86 or eAPC‐4‐1BBL using a multiplex bead assay. The bars in the column graphs indicate median values. Statistical analysis was performed using one‐way ANOVA and Bonferroni post‐test (*p ≤ 0.05; **p ≤ 0.01; ***p ≤ 0.001). All data were measured by flow cytometry.

The costimulatory receptor 4‐1BB has been previously demonstrated to play a prominent role in enhancing CD8 T‐cell responses [10, 11, 22]. Consequently, we applied our eAPC‐platform to evaluate the influence of 4‐1BB costimulation on virus‐specific human CD8 T cells. For this, eAPC expressing 4‐1BBL were generated and their stimulatory capacity was compared to eAPC expressing CD28 ligands in our in vitro culture system. Similar expression levels of HLA‐A2 and expression of costimulatory ligands on the different eAPC lines was confirmed by flow cytometry (Supporting information Fig. S2B). Stimulation cultures with K562 cells expressing HLA‐A2 (K562‐A2) and the corresponding costimulatory molecules but no viral antigens were also performed. Similar to CD86 or CD80, the presence of 4‐1BBL on the eAPC also induced a significantly higher proliferation of CD8 T cells of HLA‐A2 positive donors. The percentages of proliferated CD8 T cells in cultures with K562‐A2 cells expressing no antigens were much lower as expected, but interestingly K562‐A2 cells expressing 4‐1BBL induced significantly higher levels of CD8 T‐cell proliferation than K562‐A2 cells expressing no costimulatory ligand (Fig. 3A and B). Importantly, when we performed pHLA‐A2 tetramer stainings in cocultures with eAPC expressing 4‐1BBL, we found that a significantly lower percentage of proliferated CD8 T cells were stained positive (Fig. 3C and D). Analysis of CD3 expression indicated that the lack of pMHC‐tetramer binding was not due to downregulation of the TCR‐CD3 complex (Supporting information Fig. S3). 4‐1BBL costimulation enhanced the number of proliferated tetramer‐negative T cells, whereas the number of antigen‐specific CD8 T‐cells was not increased (Fig. 3D). High numbers of proliferated tetramer‐negative T cells were detected in stimulation cultures with eAPC‐41BBL but not with 4‐1BBL expressing cells not displaying viral antigens (K562‐A2‐4‐1BBL), indicating that 4‐1BBL stimulation promoted activation of bystander CD8 T cells (Fig. 3E). 4‐1BBL induced bystander activation was also observed in eAPC stimulation cultures with purified CD8 T cells (Supporting information Fig. S4).

In addition, we observed a massive expansion of NK cells (CD3^−^CD56^+^) in PBMCs that had been stimulated with eAPC‐4‐1 BBL. NK cells proliferation induced by K562‐A2‐4‐1BBL cells was higher compared to K562‐A2 or K562‐A2‐CD86 cells, but much lower compared to eAPC‐A2‐4‐1BBL. This indicates that massive NK cell proliferation induced by 4‐1BBL depended on the presence of T‐cell antigens (Fig 3F). In addition, we found significantly elevated levels of cytotoxic effector molecules, such as IFN‐γ, granzyme A, perforin, and granulysin, in eAPC‐4‐1BBL stimulated cultures (Fig. 3G). Together, these data suggest that costimulation via the CD28 ligands CD80/CD86 favors antigen‐specific responses, whereas 4‐1BBL promotes proliferation of bystander CD8 T cells and NK cells as well as cytotoxic effector functions. Since IL‐12 and IL‐18 have been implicated in T‐cell bystander activation, we tested the effect of combined blockade of these cytokines in our stimulation cultures. We observed a significant effect, but CD8 bystander activation and NK‐cell proliferation were still higher in eAPC‐4‐1BBL stimulation cultures compared to eAPC‐CD86 stimulation cultures (Supporting information Fig. S5).

### CD8 T‐cells proliferating in response to their antigen express elevated levels of TIM‐3 and PD‐1

**Figure 4:**
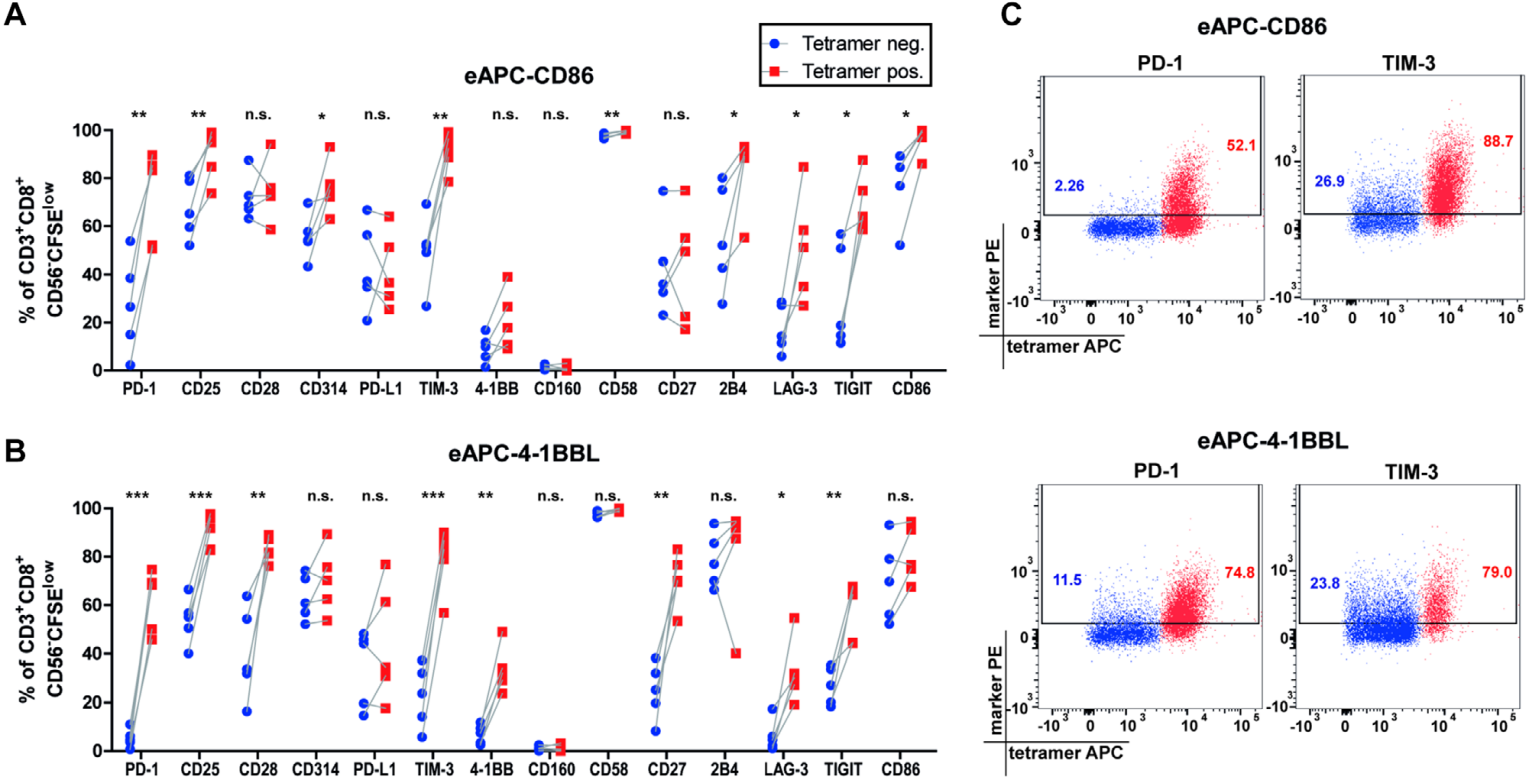
CD8 T‐cells proliferating in response to cognate antigen express elevated levels of TIM‐3 and PD‐1. (A,B) The expression of various cell surface markers related to activation, costimulation, or coinhibition on proliferated CD8 T cells after stimulation with eAPC‐CD86 (A) or eAPC‐4‐1BBL (B) was assessed in 5 independent experiments using one donor per experiment. The percentages of marker positive cells within the pHLA‐A2 tetramer‐positive and ‐negative CD8^+^CFSE^low^ T cells were compared. (C) Representative dot plots of these five independent experiments showing the expression of PD‐1 and TIM3 after stimulation with eAPC‐CD86 or eAPC‐4‐1BBL. Statistical analysis was performed using a paired *t*‐test (*p ≤ 0.05; **p ≤ 0.01; ***p ≤ 0.001). All data were measured by flow cytometry.

Next, we investigated whether CD8 T cells responding to cognate antigen and bystander T cells would differentially express markers associated with activation, costimulation, or coinhibition. For this, PBMC were stimulated for 7 days with eAPC‐CD86 or eAPC‐4‐1BB, then stained with the pHLA‐A2 tetramer pool and a panel of antibodies including i.a. CD25, CD27, CD28, PD‐1, TIM‐3, and LAG‐3. Both types of eAPCs induced increased expression of several markers in proliferated T cells that stained positive for the pHLA‐A2‐tetramer pool (Fig. 4A and B). This difference was most pronounced for PD‐1 and TIM‐3, which were absent from the majority of tetramer negative CD8 T cells but highly expressed on pHLA‐A2‐tetramer positive T cells (Fig. 4C). Besides, when comparing CD86‐ and 4‐1BBL costimulation, the expression of 4‐1BB and CD27 was higher on antigen‐specific T cells that had proliferated in response to 4‐1BBL. CD86 costimulation induced higher PD‐1 expression on antigen‐specific T cells as well as tetramer‐negative T cells (Supporting information Fig. S6).

### The 4‐1BB agonist urelumab promotes antigen independent T‐cell responses and NK‐cell expansion

**Figure 5:**
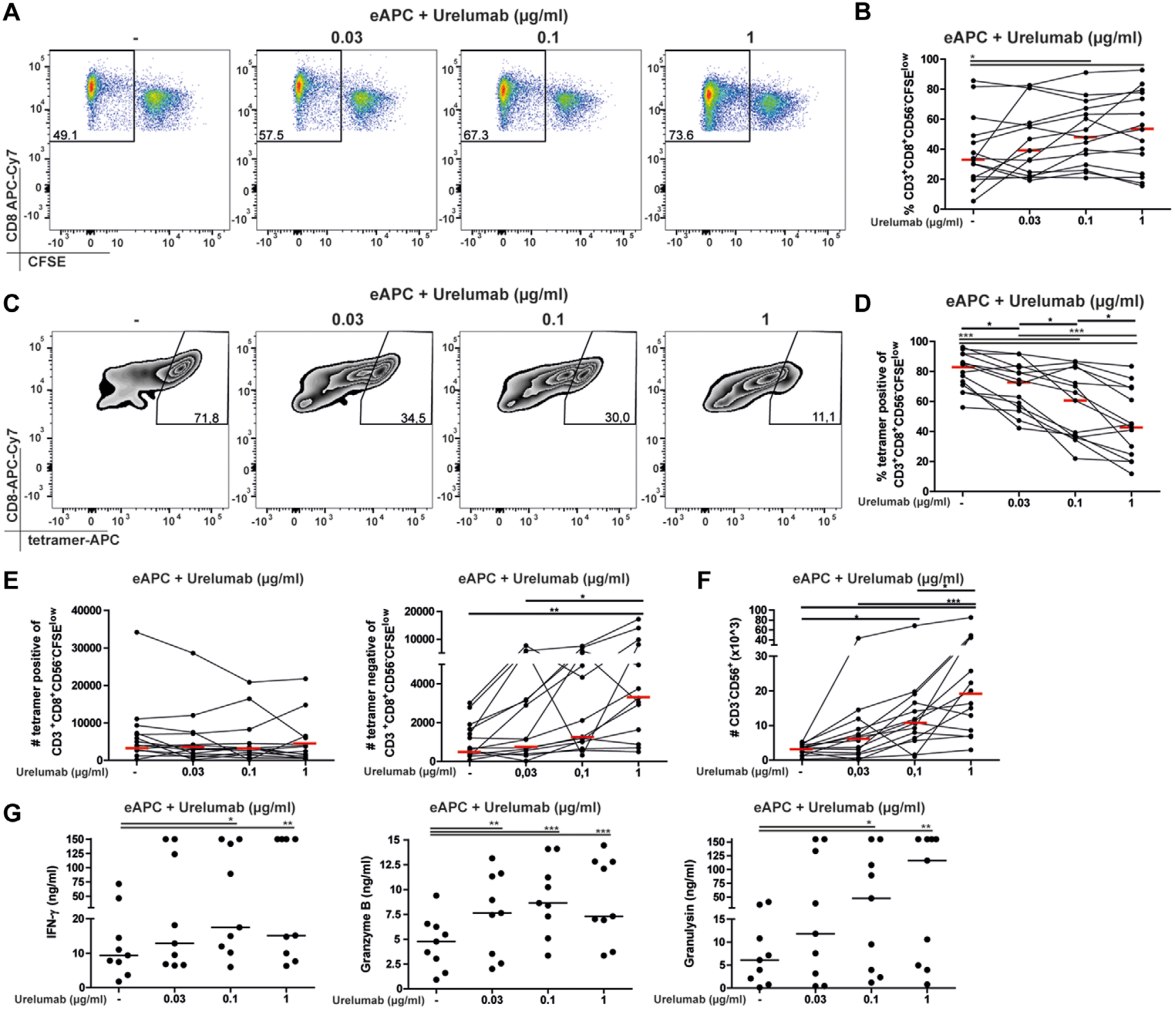
The 4‐1BB agonist urelumab promotes bystander T‐cell responses and NK‐cell expansion. (A,B) Proliferation of CD8 T cells in response to eAPC in the presence of different concentrations of the agonistic therapeutic 4‐1BBL antibody urelumab. (A) One representative experiment and (B) cumulative data of 11 different donors obtained in six independent experiments with one to two donors per experiment are shown. (C) Staining of proliferated CD8 T cells with a pHLA‐A2 tetramer pool of one representative donor. (D) Percentages of pHLA‐A2 tetramer pool positive CD8 T cells, (E) absolute numbers of tetramer pool positive or tetramer pool negative CFSE^low^ CD8 T cells and (F) NK cells are shown from 11 different donors obtained in six independent experiments with one to two different donors. (A‐F) All data were measured by flow cytometry. (G) Concentrations of IFN‐γ, granzyme A, and granulysin in the supernatants of PBMC from 9 different donors obtained in six independent experiments with one to two different donors cocultured for 7 days with eAPC in the presence of different concentrations of urelumab measured by flow cytometry using the LEGENDplex human CD8/NK panel. The bars in the column graphs indicate median values. Statistical analysis was performed using one‐way ANOVA, followed by Bonferroni post‐test (*p ≤ 0.05; **p ≤ 0.01; ***p ≤ 0.001).

The unexpected propensity of 4‐1BB costimulation to promote bystander T‐cell activation prompted us to investigate the effects of urelumab, a 4‐1BB agonist in our in vitro stimulation system. PBMCs were stimulated with eAPC in the presence of different concentrations of this antibody. Urelumab enhanced T‐cell proliferation in some donors but overall the percentages and numbers of proliferated CD8 T cells were not significantly higher in our data set (Fig. 5A and B). Importantly, the presence of this antibody reduced the percentage of proliferated CD8 T cells that stained positive for the pHLA‐A2 tetramer pool and significantly increased the numbers of tetramer‐negative proliferated CD8 T cells, indicating proliferation independent of the eAPC expressed antigens (Fig. 5C to E). Significantly higher numbers of tetramer‐negative proliferated CD8 T cells were detected, demonstrating that urelumab induced bystander proliferation (Fig. 5E). These results indicate that the engagement of 4‐1BB by 4‐1BBL or by urelumab induces bystander activation of T cells. In line, we observed that 4‐1BBL and urelumab induced strong NF‐kB activation in absence of TCR/CD3 signals in 4‐1BB‐expressing Jurkat T‐cell reporters (Supporting information Fig. S7). Similar to 4‐1BBL expressed on eAPC, urelumab promoted a massive expansion of NK cells and the production of cytotoxic effector molecules including IFN‐γ, granzyme B, and granlysin (Fig. 5F and G). Moreover, urelumab also induced bystander proliferation upon CD28‐costimulation, as it significantly reduced the percentage of pHLA‐A2 tetramer positive CFSE^low^ CD8 T cells in stimulation cultures with eAPC‐CD80 (Supporting information Fig. S8).

### CD27 signals did not promote expansion of bystander CD8 T cells in vitro

**Figure 6:**
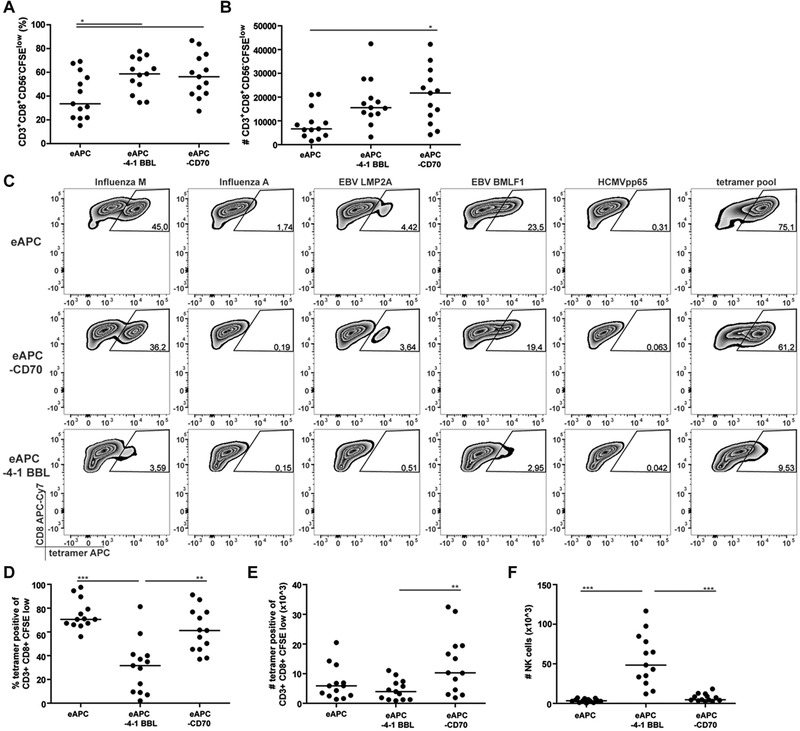
4‐1BB and CD70 have divergent effects on the expansion of antigen‐specific CD8 T cells and NK cells. (A,B) Proliferative responses of CD8 T cells in PBMC from 13 different donors stimulated in seven independent experiments with one to two donors with eAPC, eAPC‐4‐1BB, or eAPC‐CD70.(A) The percentages and (B) absolute numbers of proliferating CD8 T cells are shown. (C) Flow cytometry contour plots of one donor showing staining of proliferated CD8 T cells with the indicated pHLA‐A2 tetramers and a pHLA‐A2 tetramer pool after stimulation with eAPC, eAPC‐4‐1BB, or eAPC‐CD70 (data are representative for samples from 13 donors analyzed in 7 independent experiments with one to two donors). (D) Percentage of pHLA‐A2 tetramer pool positive cells among CD8 T cells, (E) Total number of tetramer positive CD8 T cells that had proliferated in response to the indicated eAPC and (F) the number of NK cells (CD56^+^CD3^−^) in stimulation cultures with the indicated eAPC. (C‐F) Data represent seven independent experiments with 13 different donors and one to two donors per experiment. The bars in the column graphs indicate median values. Statistical analysis was performed using one‐way ANOVA, followed by Bonferroni post‐test (*p ≤ 0.05; **p ≤ 0.01; ***p ≤ 0.001). All data were measured by flow cytometry.

CD27 and 4‐1BB are both members of the TNFRSF. Similar to 4‐1BB, CD27 is targeted in clinical trials to enhance antitumor T‐cell immunity in cancer patients. The presence of the CD27‐ligand CD70 on eAPC resulted in a higher percentage of CFSE^low^ CD8 T cells and in significantly enhanced numbers of proliferated CD8 T cells (Fig. 6A and B). However, in contrast to 4‐1BBL costimulation, the activation of CD8 T cells in the presence of CD70 did not cause significantly reduced percentages of pHLA‐A2 tetramer positive cells, but instead resulted in significantly higher absolute numbers of tetramer positive proliferated CD8 T cells (Fig. 6C to E). Moreover, although NK cells express CD27 [23], the number of NK cells was not significantly increased in cultures stimulated with eAPC‐CD70 (Fig. 6F).

## Discussion

Accessory signals play a decisive role in the response of T cells that encounter antigens. The blocking of coinhibitory receptors, such as PD‐1, has been demonstrated to efficiently enhance antitumor response. However, only a subset of tumor patients is able to benefit from treatment with antibodies targeting PD‐1 or its major ligand PD‐L1. Consequently, intense research efforts try to exploit additional immune checkpoints to combat cancer [24, 25, 26]. Numerous antibodies targeting additional inhibitory receptors, such as LAG‐3, VISTA and TIM‐3, are currently being tested in clinical trials [27, 28, 29]. Another powerful means to potentiate antitumor responses is the use of agonistic antibodies to costimulatory receptors. Therefore, the current focus lies on antibodies against TNFRSF members, such as 4‐1BB, CD27, OX40, and GITR [30, 31, 32].

Studies in numerous preclinical models have suggested that 4‐1BB agonists can promote antitumor immunity when used alone or in combination [33, 34, 35, 36]. However, systemic 4‐1BB stimulation is also associated with undesired side‐effects. In mice, the administration of 4‐1BB agonistic antibodies has been shown to cause polyclonal activation of CD8^+^ T cells, secretion of inflammatory cytokines, and immune‐related anomalies that impaired the function of the spleen, bone marrow, and liver [37, 38, 39]. In initial clinical studies, urelumab was found to cause severe liver toxicity, leading to the termination of these trials and the administration of lower doses in subsequent studies [31, 40]. Extensive research has been performed to understand the mechanisms of 4‐1BB agonist‐induced side‐effects. In BALB/c and C57BL/6 mice, treatment with 4‐1BB agonists led to mononuclear inflammation driven by antigen‐independent activation of nontumor‐specific CD8 T‐cells activation [38, 39]. Moreover, in an earlier study, it had been reported that 4‐1BB stimulation delivers an antigen‐independent growth signal for murine T cells with a memory phenotype [41]. To our knowledge, it has not been investigated whether 4‐1BB also promotes antigen‐independent proliferation of human CD8 T cells.

Here, we describe the generation of a novel system of eAPC for the evaluation of CD8 T‐cell responses to antigen‐specific stimulation in vitro. Our eAPC express multiple viral epitopes and thus induce substantial CD8 T‐cell responses in the majority of donors. By using a pool of MHC‐class I tetramers presenting these epitopes, we were able to assess the contingent of antigen‐specific CD8 T cells that proliferated in response to these eAPC. Our results demonstrated that 4‐1BB costimulation enforced proliferation of CD8 T cells not reactive with the antigens presented by the eAPC. Importantly, this expansion depended on the response of antigen‐specific CD8 T cells, indicating that 4‐1BB costimulation promotes bystander activation of CD8 T cells. Moreover, we observed that the presence of 4‐1BBL on eAPC induced massive NK‐cell proliferation. Previous work has established that 4‐1BBL potently augment NK‐cell expansion and effector function mediated by cytokines such as IL‐15 and IL‐21 [42, 43, 44]. Our results indicate that factors expressed by activated T cells also induce massive NK‐cell expansion in the presence of 4‐1BBL. Importantly, we show that the 4‐1BB agonistic antibody urelumab also strongly promotes bystander proliferation of CD8 T cells and expansion of NK cells. Further studies will be required to identify the signals that cooperate with 4‐1BB to mediate potent proliferation and effector cytokine production by CD8 T cells and NK cells.

Our efforts to identify markers that would distinguish CD8 T cells that had proliferated in response to eAPC‐expressed epitopes from CD8 T cells that had proliferated in the absence of cognate antigens revealed an upregulation of several markers including PD‐1 and TIM‐3. These receptors have been implicated in T‐cell exhaustion, but also mark activated T cells. CD25, a bona fide T‐cell activation marker, was also upregulated in the tetramer‐reactive subset, indicating that the antigen‐specific T cells are characterized by a higher state of activation than bystander‐activated T cells.

Similar to 4‐1BB, CD27 is also considered a promising target for cancer immunotherapy [45]. Two CD27 agonists have already entered clinical trials with patients suffering from malignancies including lymphomas, melanoma, and renal cell carcinoma [32]. We found that eAPC expressing the CD27 ligand CD70 strongly promoted tetramer‐positive CD8 T‐cell proliferation but not bystander CD8 T‐cell responses.

In summary, we have devised a novel engineered antigen presentation platform for the ex vivo stimulation of human CD8 T cells. Our study revealed a previously unappreciated feature of 4‐1BB, namely that, in the presence of 4‐1BB signals, antigen‐specific human CD8 T‐cell responses are accompanied by bystander activation of immune cells. Apparently, this is not a general effect of costimulation via TNFR‐SF members, since triggering of CD27 did not significantly enhance the bystander activation of human T cells or NK‐cell expansion. The propensity of costimulation agonists to promote off‐target immune activation when administered alone or in combination with other immunomodulatory molecules should be taken into account when devising therapeutic strategies to enhance immune responses against cancer cells. Engineered antigen presentation platforms, like the one presented here, are powerful tools to both assess the effects of immunostimulatory antibodies on antigen‐specific immune response and identify undesired off‐target effects.

## Material and methods

### Human subjects

This study was approved by the Ethics Committee of the Medical University of Vienna (EK110/2011; 1183/2016). Peripheral blood was obtained from healthy donors after written informed consent was obtained.

### Cell culture

All cell lines were cultured in RPMI 1640 medium (Gibco, Thermo Fisher Scientific) supplemented with 2 mM l‐glutamine, 100 U/mL penicillin, 100 μg/mL streptomycin, and 10% of fetal calf serum (Sigma Aldrich, St. Louis, MO). For authentication, cell lines were stained with a panel of antibodies. All cell lines were tested for absence of mycoplasma using a method described recently [46].

### Generation of eAPC

Cell lines expressing HLA‐A2 alone or coexpressing HLA‐A2 and the human costimulatory ligands CD80, CD86, 4‐1BBL, or CD70 were generated by retroviral transduction of the K562 cell line, a human erythroleukemia cell line (ATCC® CCL‐243, Manssas, VA), in conjunction with flow sorting. To generate eAPC or eAPC expressing costimulatory ligands, K562 cell clones with high levels of expression of these molecules were selected and were transduced with a retroviral construct encoding five major HLA‐A2 restricted antigenic peptides (GILGFVFTL‐Influenza M; FMYSDFHFI‐Influenza A; CLGGLLTMV‐EBV‐LMP2A; GLCTLVAML‐EBV‐BMLF1; NLVPMVATV‐HCMVpp65) linked via a GGS_3_ peptide linker to eCFP and a PEST sequence [47] for efficient proteasome targeting and antigen processing. To analyze the proteasome targeting of this construct, eAPC were treated for 16 h with proteasome inhibitor MG115 (1 μM; Sigma Aldrich) and analyzed for eCFP expression by flow cytometry.

### T‐cell reporter assays

A previously described Jurkat NFκB::eGFP reporter cell line [48] was engineered to express a TCR specific for the CMV‐pp65 antigen (NLVPMVATV) [49]. The resulting reporter cells were cocultured with the eAPC at a ratio of 1:1 for 24 h. The expression of eGFP in the reporter cell lines was assessed by flow cytometry using a FACSCanto II (BD Bioscience, Franklin Lakes, NJ). Previously described Jurkat‐triple parameter reporter cells expressing 4‐1BB or control‐triple parameter reporter cells were cultured with or without T‐cell stimulator cells (TCS) at a ratio of 2.5:1. Urelumab (purchased from Creative Biolabs, Shirley, NY) was added at the indicated concentrations [50]. In additional experiments, these reporter cells were cocultured with K562‐HLA‐A2, K562‐HLA‐A2‐CD86, or K562‐HLA‐A2‐4‐1 BBL. After 24 h of stimulation, the expression of the NF‐kB reporter molecule eCFP was analyzed by flow cytometry using a LSR II Fortessa (BD Bioscience).

### Antigen‐specific stimulation of PBMCs

PBMCs from heparinized whole blood samples of HLA‐A2 positive donors were isolated by gradient density centrifugation using Ficoll‐Paque PLUS (GE Healthcare, Chicago, IL). HLA‐A2 positive samples were identified by staining with a HLA‐A2 specific antibody (#BB7.2, BioLegend, San Diego, CA) and flow cytometry. The PBMC of HLA‐A2 positive donors were labeled with 1 mM of CFSE (Invitrogen, Thermo Fisher Scientific) or eFluor 670 (Affymetrix, Thermo Fisher Scientific). K562‐HLA‐A2 and eAPC cell lines were irradiated (120 Gy) and added at a ratio of 1:5 to the PBMC, then cultivated in AIM‐V (Gibco) supplemented with 2% human AB serum (Sigma). Antigenic peptides (used at a final concentration of 0.3 μg/mL each; purchased from Peptide 2.0,) were added at culture onset as indicated. The agonistic 4‐1BB antibody urelumab was added to some cultures at indicated concentrations. Following 7 days of culture, the cells were harvested, stained with antibodies and HLA‐A2/p tetramers and subjected to flow cytometric analysis. Absolute cell counts were determined by adding counting beads beads (2 × 10^4^ 1,2,3 counting beads, Thermo Fisher) to each sample. The culture supernatants were collected and stored at –20°C. All experiments were performed in duplicate.

For the restimulation experiments, eFluor670‐labelled PBMC stimulated for 7 days with K562‐HLA‐A2 or eAPC as described above, were harvested, labeled with CFSE, and stimulated with irradiated K562‐HLA‐A2 cells or eAPC for another 7 days, before being subjected to flow cytometric analysis, as described above.

### Flow cytometric analysis

Flow cytometry experiments were performed in adherence to the guidelines published by Cossarizza at al. [51]. After blocking of the harvested cells from the coculture experiments for 20 min at 4°C with 20% AB‐serum in PBS, the cells were stained for 30 min with fluorophore‐labeled antibodies and pHLA‐A2 tetramers at 4°C. The following antibodies were used: CD80 PE (2D10), CD86 PE (IT2.2), 4‐1BBL PE (5F4), CD70 PE (113‐[16]), CD3 BV510 (UCHT1), CD4 BV421 (RPA‐T4), CD8 APC‐Cy7 (HIT8a), HLA‐A,B,C APC (W6/32), and CD56 PE‐Cy7 (CMSSB).

APC‐labeled MHC class I‐peptide tetramers (HLA‐A‐02:01/GILGFVFTL; HLA‐A‐02:1/FMYSDFHFI; HLA‐A‐02:01/CLGGLLTMV; HLA‐A‐02:01/NLVPMVATV; HLA‐A‐02:01/GLCTLVAML, and HLA‐A‐02:01/SLLQHLIGL used as a negative control) were kindly provided by the NIH Tetramer Core Facility. Flow cytometry was performed using a FACS Canto II (BD Bioscience). FlowJo software (Tree Star, Inc., San Carlos, CA) was used for data analysis.

### Assessment of cytotoxicity

The PBMC of the HLA‐A2 positive donors stimulated with eAPC‐CD80 cells for 7 days as described above, were flow‐sorted for proliferated CD8^+^CD56^‐^ cells. The isolated cells were then cocultured with irradiated (120 Gy) K562‐HLA‐A2‐CD80 and eAPC‐CD80 cell lines overnight at the indicated ratios. Subsequently, the cells were harvested and stained with CD8 and CD80 antibodies to identify CD8 T cells and K562‐A2‐CD80/eAPC‐CD80, respectively, by flow cytometry; Viable K562‐HLA‐A2 cells and eAPC were identified by their FSC/SSC properties.

### Cytokine analysis

The supernatants of the PBMC‐eAPC cocultures were harvested at day 7, pooled from duplicate wells and stored at −20 °C. IL‐2, IL‐4, IL‐10, IL‐6, IL‐17A, TNF‐α, sFas, sFasL, IFN‐γ, granzyme A, granzyme B, perforin, and granulysin were determined using the LEGENDplex human CD8/NK panel (13‐plex, Biolegend) according to the manufacturer's instructions. The cytokine content of each donor is represented by a single dot.

### Statistical analysis

GraphPad Prism 7 (GraphPad Software Inc., La Jolla, CA) was used to perform the statistical analyses. One‐way repeated measurement ANOVA was performed to analyze the proliferation, cell counts, tetramer positivity, and cytokine data. Comparisons between the groups were performed using one‐way ANOVA and Bonferroni post‐hoc test. *p* values under 0.05 were considered significant (*), *p* ≤ 0.01, (**), *p* ≤ 0.001, (***).

## Conflict of interest

All authors declare to have no commercial or financial conflict of interest.

## Author contributions

MR, PS, and BJS designed the research and wrote the manuscript. MR performed the experiments and analyzed data. SR, JL, and CB generated and validated the constructs and cell lines. BB contributed to the research design. All authors contributed to the writing and revision of the manuscript.

### Peer review

The peer review history for this article is available at https://publons.com/publon/10.1002/eji.202048762.

**Abbreviations**
AICD: activation‐induced cell death
CTL: cytotoxic T lymphocytes
eAPC: engineered antigen presenting cell
sFas: soluble Fas
TIL: tumor‐infiltrating lymphocyte
TNFRSF: TNF receptor superfamily

## Supporting information



Supporting Information

## Data Availability

The data that support the findings of this study are available from the corresponding author upon reasonable request.
