# Supplementary material for: 4‐1BB costimulation promotes bystander activation of human CD8 T cells
Source: Eur J Immunol. 2020 Dec 23;51(3):721–33. doi: 10.1002/eji.202048762 (PMC7986150; doi:10.1002/eji.202048762)
Supplement: Supplementary file 1 — Supporting Information [file EJI-51-721-s001.pdf]

Supplementary data

4-1BB costimulation promotes bystander activation of human CD8 T cells

Manuel Reithofer, Sandra Rosskopf, Judith Leitner, Claire Battin, Barbara Bohle, Peter Steinberger and Beatrice Jahn-Schmid

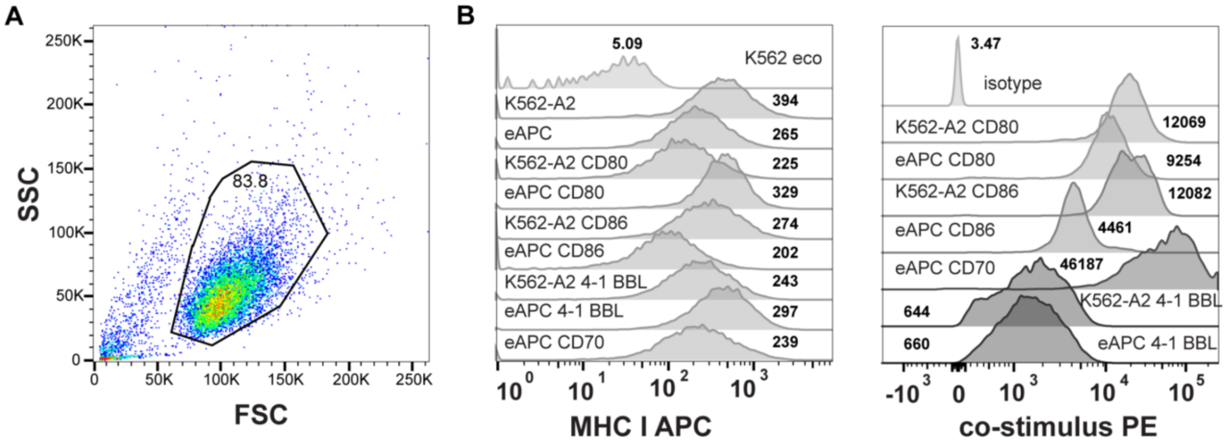

Figure S1. Evaluation of eAPC. (**A**) Gating strategy for K562 and eAPC. (**B**) K562 cells expressing HLA-A2 and K562 cells co-expressing HLA-A2 and the indicated co-stimulatory ligands were probed with a APC-labeled MHC class I specific antibody (clone W6/32; left) or PE-labeled antibodies to the respective costimulatory ligands (right). The parental K562 line (left) and K562-A2 stained with isotype control (right) were used as negative controls. The numbers indicate gMFI values. All data were obtained by flow cytometry.

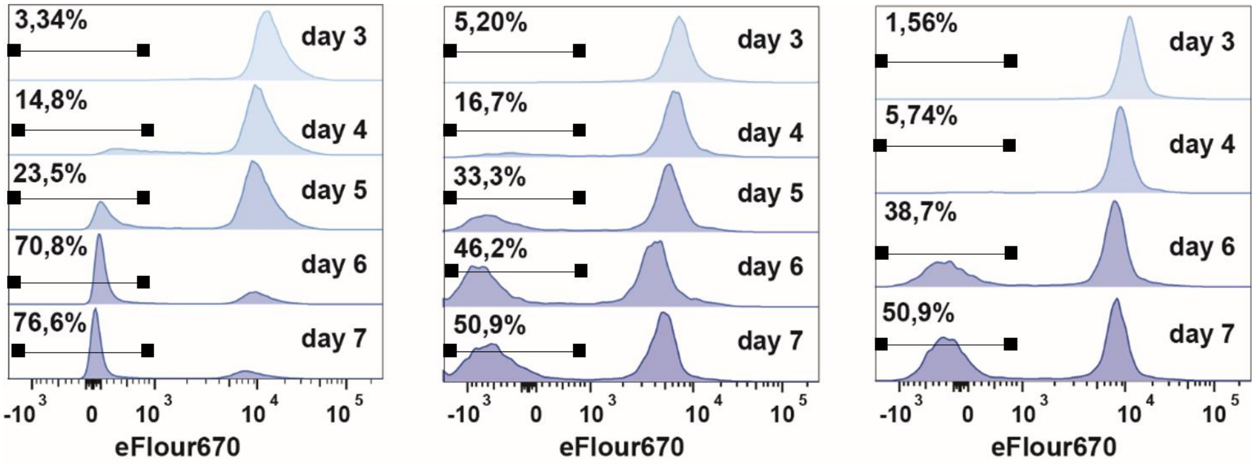

Figure S2. Time course of proliferation of CD8 T cells upon stimulation with eAPC. eFluor670 labelled PBMC were stimulated with eAPC-CD80 and fluorescent dye dilution in the CD8 T cell subset was measured at the indicated time points. Data represent three independent experiments with 3 different donors. All data were obtained by flow cytometry.

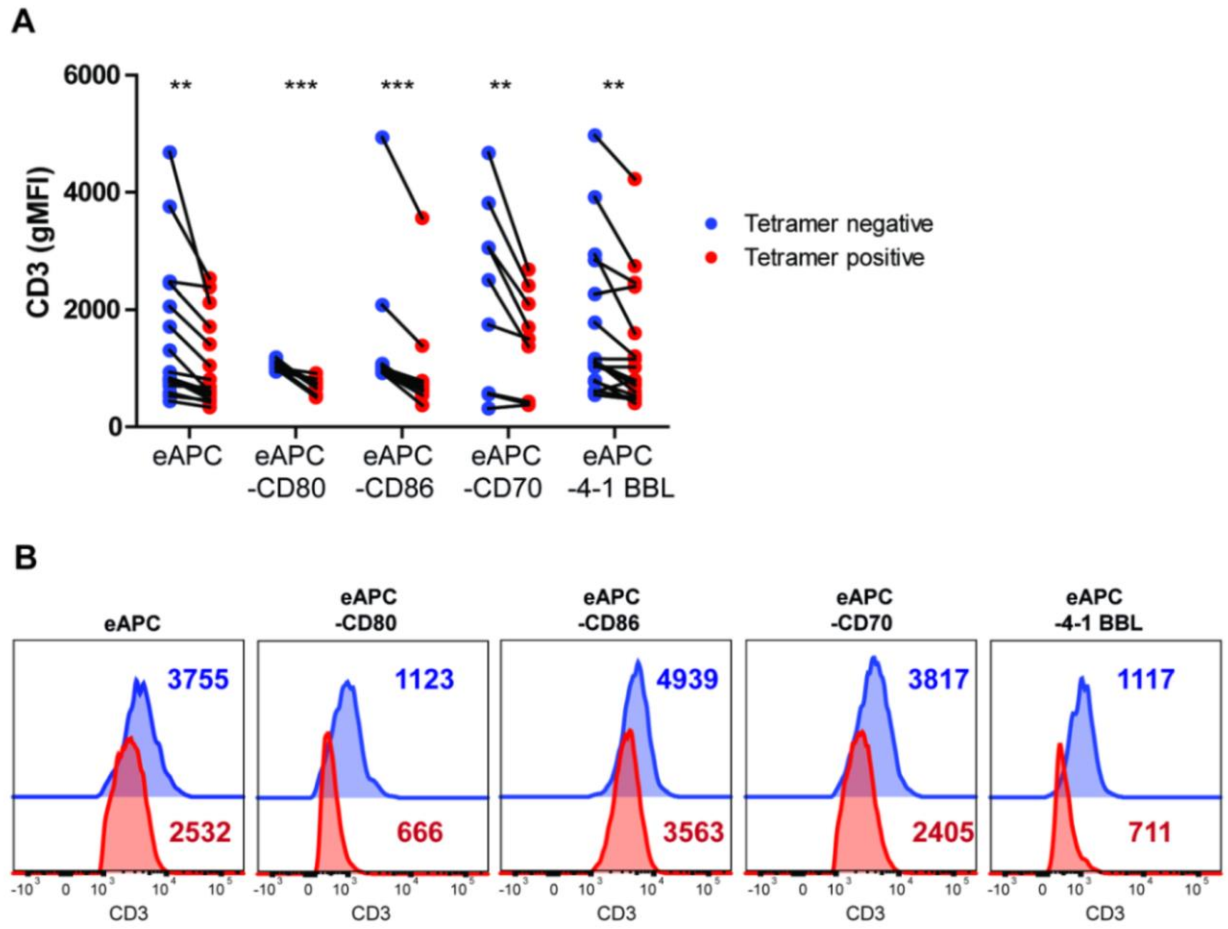

Figure S3. CD3 expression of proliferated tetramer-negative CD8 T cells. (**A**) Comparison of the gMFI values in the pMHC tetramer-negative and pMHC tetramer-positive proliferated ($CFSE^{low}$) CD8 T cells after 7 days of stimulation with the indicated eAPC. (**B**) CD3 expression of representative donors is shown as histograms for the pMHC tetramer negative (blue) and pMHC tetramer positive (red) $CFSE^{low}$ CD8 T cells. The numbers indicate gMFI values. All data were obtained by flow cytometry. Statistical analysis was performed using a paired t-test (**P ≤ 0.01; ***P ≤ 0.001).

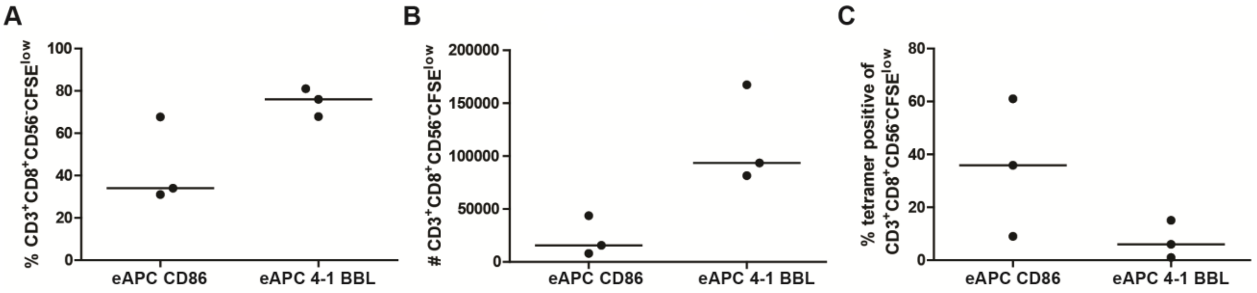

Figure S4. Effects of CD86 and 4-1BBL co-stimulation on isolated CD8 T cells. CD8 T cells were isolated using a CD8 T Cell Isolation Kit (Miltenyi Biotec, Bergisch Gladbach, GER), CFSE labelled and thereafter stimulated with the indicated eAPC for 7 days. Percentage (**A**) and numbers (**B**) of proliferated CD8 T cells and (**C**) percentages of pHLA-A2 tetramer pool positive proliferated CD8 T cells from three independent experiments with three different donors performed are shown. All data were obtained by flow cytometry.

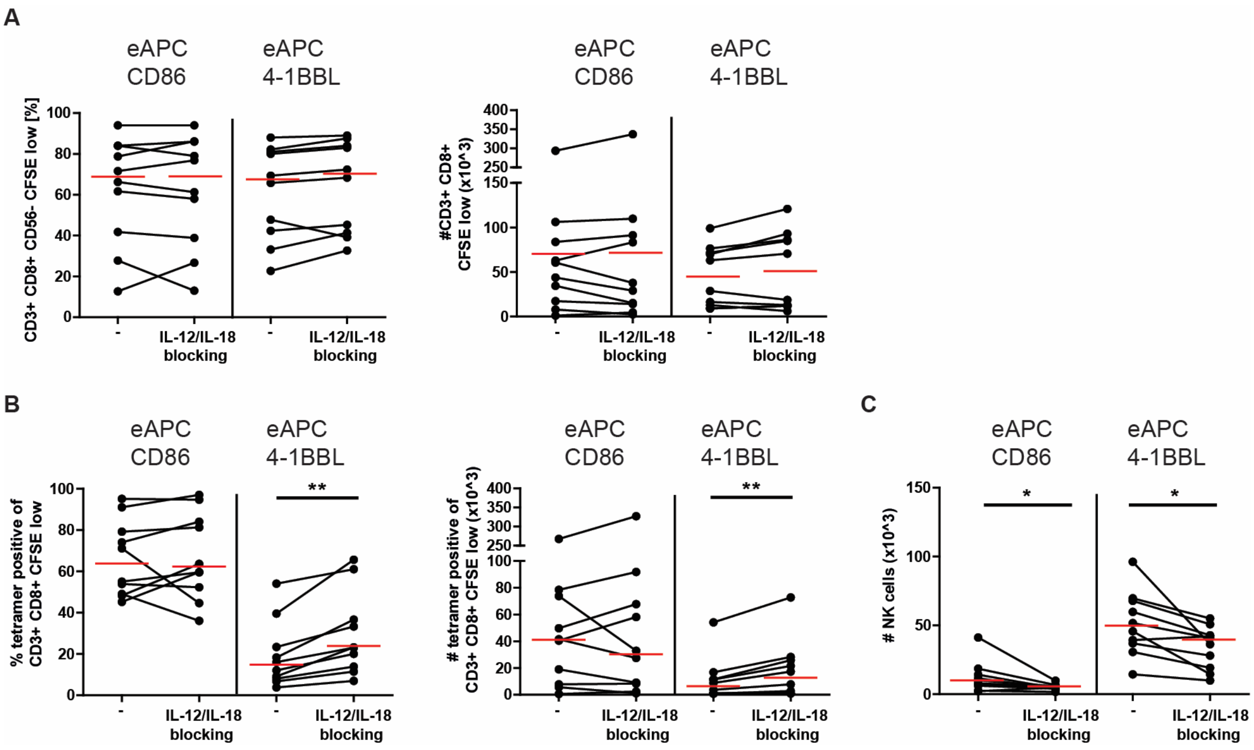

Figure S5. Effects of combined IL-12/IL-18 blockade on T cell bystander activation. Stimulation of PBMC was performed for 7 days with the indicated eAPC in the presence of blocking antibodies for IL-12 (30 µg/ml, Stelara, Janssen-Cilag GmbH, Neuss, GER), IL-18 (10 µg/ml, MBL, Nagoya, JPN) and IL18R (10 µg/ml, Biolegend, San Diego, CA, USA) or without antibodies. (**A**) Proliferation of CD8 T cells is shown as percentage (left) or absolute cell counts (right). The dots represent the proliferation of CD8 T cells from individual donors in response to stimulation with the indicated eAPC. (**B**) Antigen specificity of proliferated, i.e. eFlour670^{low} CD8 T cells, was determined by staining with a pool of all five pHLA-A2 tetramers and shown as percentage (left) or absolute cell counts (right). The dots represent the proliferation of CD8 T cells from in individual donors in response to stimulation with the indicated eAPC. (**C**) Counts of NK cells upon PBMC stimulation with the respective eAPC are depicted. (A-C) Data are derived from 10 different donors obtained in 4 independent experiments with 1-3 donors. Red bars indicate median values. All data were obtained by flow cytometry. Student´s t-test (*P ≤ 0.05; **P ≤ 0.01).

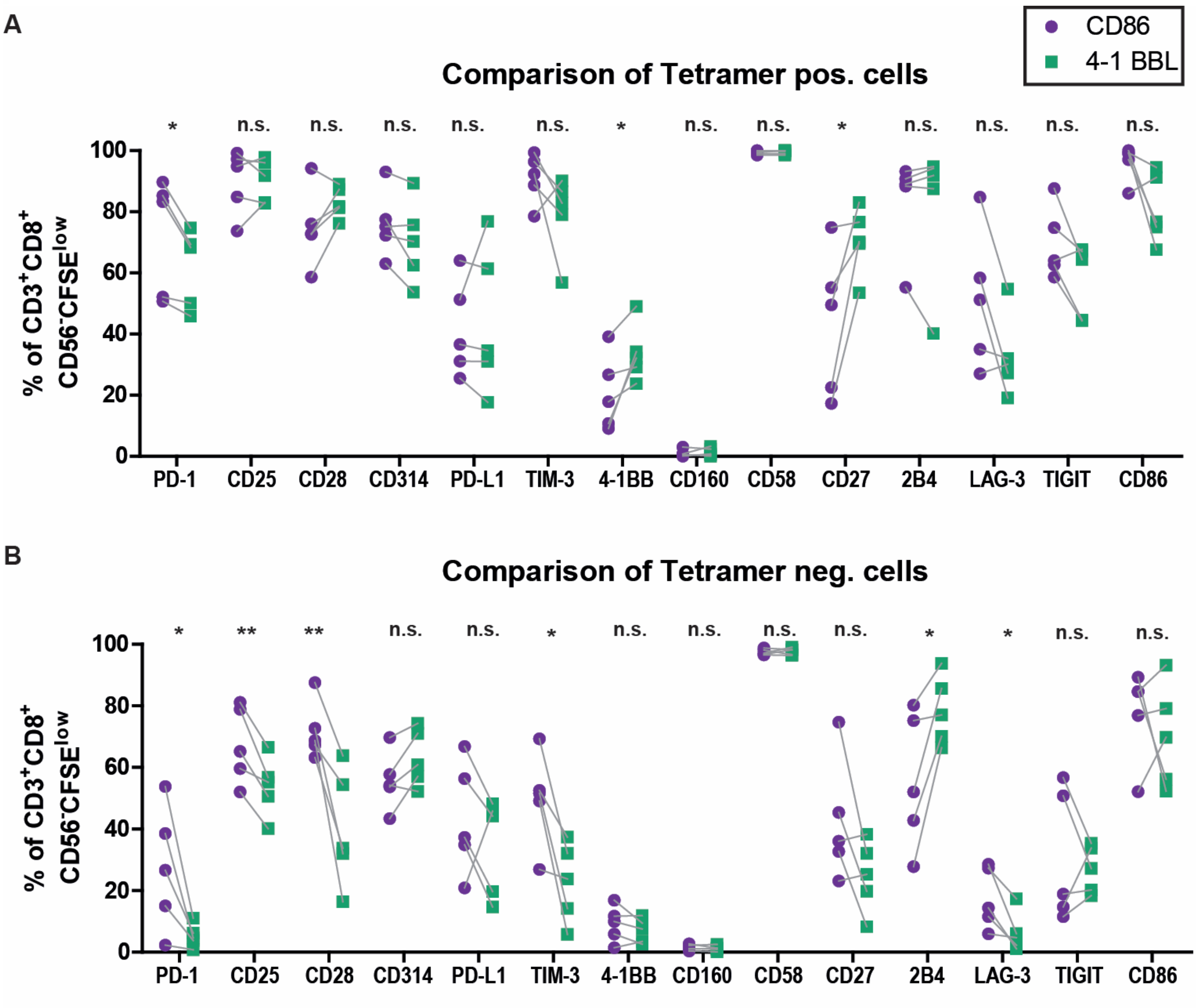

Figure S6. CD8 T cells proliferating in response to cognate antigen express elevated levels of TIM-3 and PD-1. The expression of various cell surface markers related to activation, costimulation or coinhibition on proliferated CD8 T cells after stimulation with eAPC-CD86 (**A**) or eAPC-4-1BBL (**B**) was assessed in 5 independent experiments with different donors. Shown are % of positive cells in (**A**) pMHC tetramer pool positive or (**B**) pMHC tetramer pool negative $CD8^{+}CFSE^{low}$ T cells. All data were obtained by flow cytometry. Paired t-test (*$P \leq 0.05$; **$P \leq 0.01$).

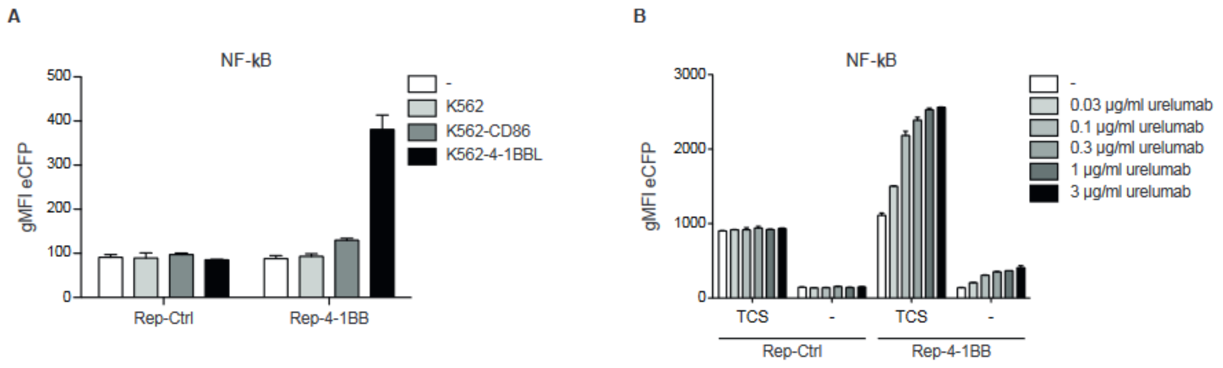

Figure S7. Engagement of 4-1BB induces TCR/CD3 independent NF-κB activation in T cell reporters. (**A**) 4-1BB expressing transcriptional reporter cells and control reporter cells were left unstimulated or were co-cultured with control-K562 cells or K562 cells expressing CD86 or 4-1BBL. After 24 h of stimulation, NF-κB reporter gene (eCFP) expression was analyzed by flow cytometry. (B) T cell reporter cells were cultured in the presence or absence of T cell stimulator cells (TCS), which induce reporter activation via the TCR/CD3 complex. Urelumab was added to the cultures at the indicated concentrations. After 24 h and NF-κB reporter gene (eCFP) expression was analyzed by flow cytometry. Data is representative of two independent experiments performed in triplicate.

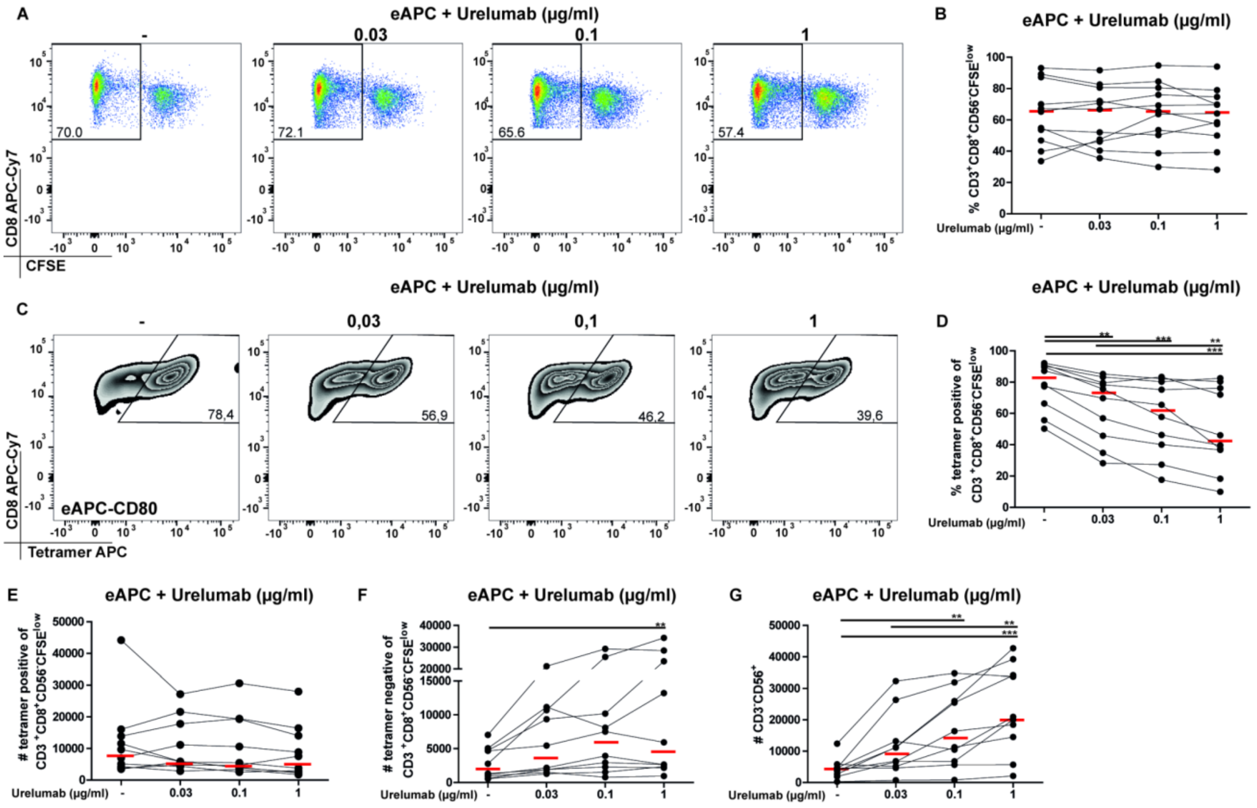

Figure S8. Effects of urelumab in presence of CD28 costimulation. Proliferation of CD8 T cells in response to eAPC-CD80 in the presence of different concentrations of urelumab. Flow cytometry dot plots of one representative experiment (**A**), and cumulative data from 10 independent experiments (**B**) are shown. (**C**) Flow cytometry contour plots showing staining of proliferated CD8 T cells with a pHLA-A2 tetramer pool after the stimulation of PBMCs of a representative donor with eAPC-CD80 in the presence of different concentrations of urelumab. (**D**) Percentages and (**E**) numbers of pHLA-A2 tetramer pool positive CD8 T cells from 10 donors. (**F**) Numbers of pHLA-A2 tetramer pool negative proliferated CD8 T cells from 10 donors. (**G**) Numbers of NK cells from 10 donors. (A-G) cumulative data of 10 different donors obtained in 6 independent experiments with 1-2 donors per experiment are shown. All data were obtained by flow cytometry. Statistical analysis was performed using one-way ANOVA followed by Bonferroni post-test (*$P \leq 0.05$; **$P \leq 0.01$; ***$P \leq 0.001$).
